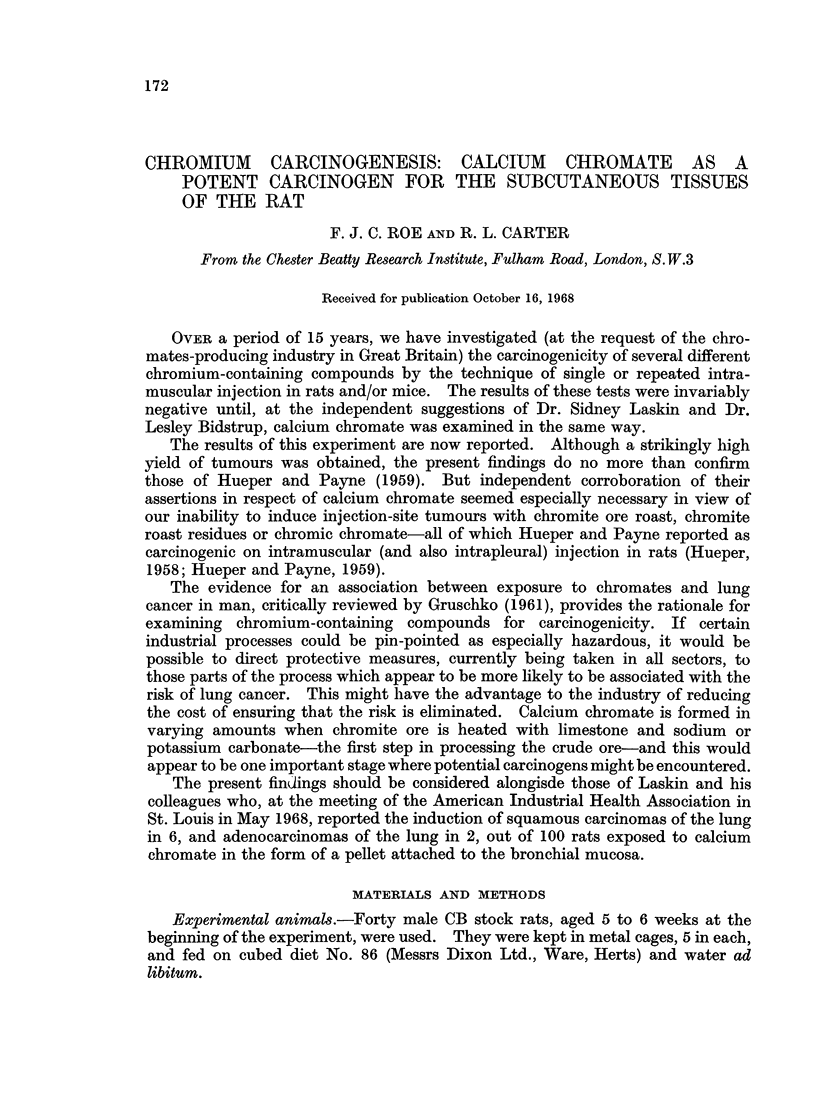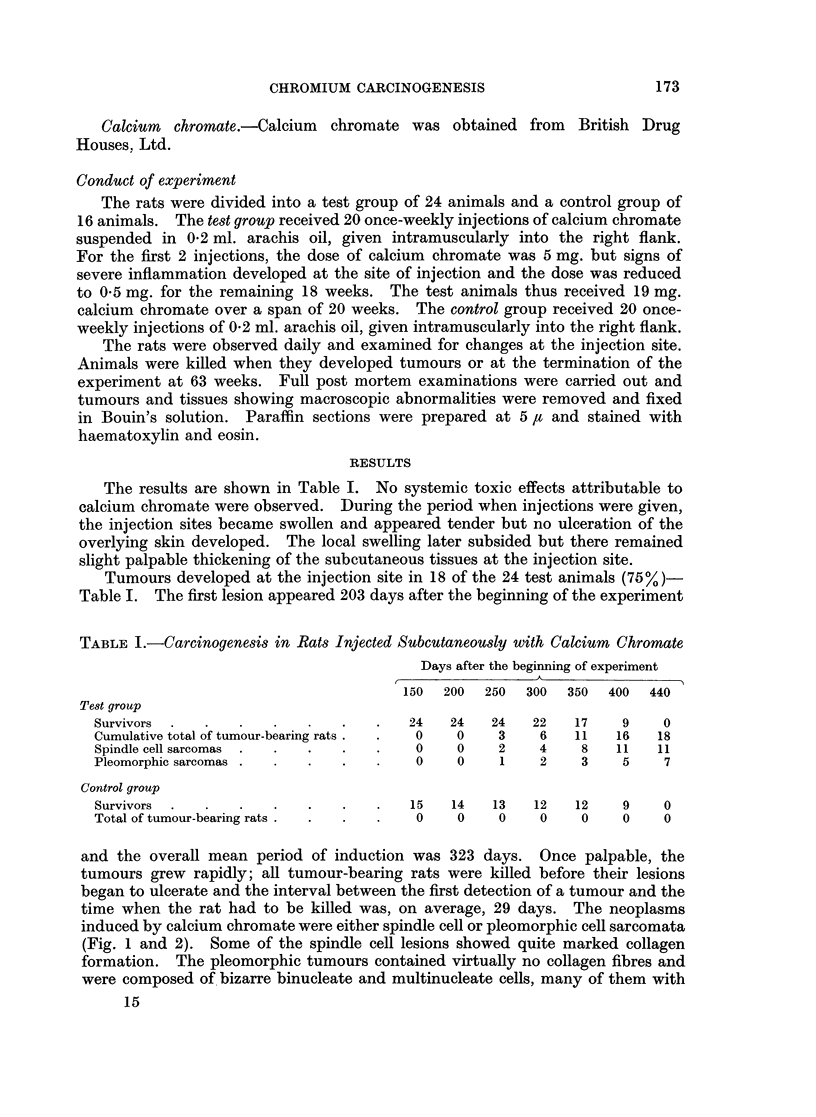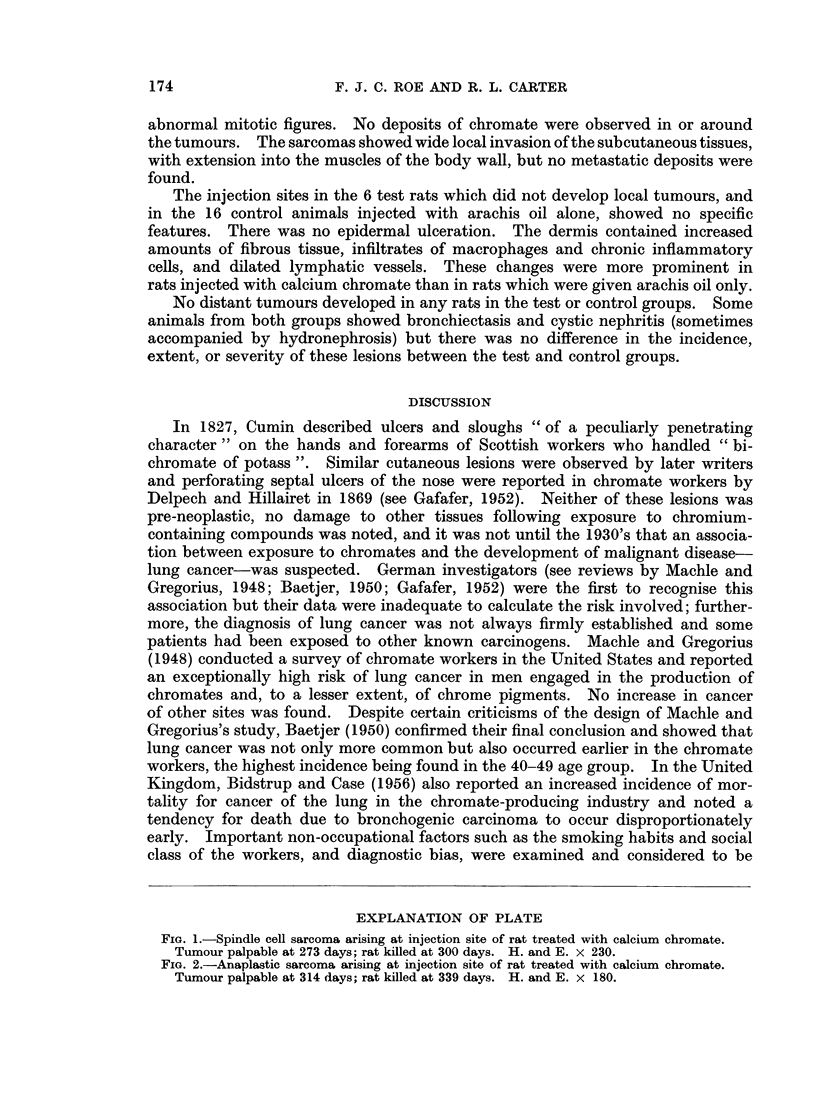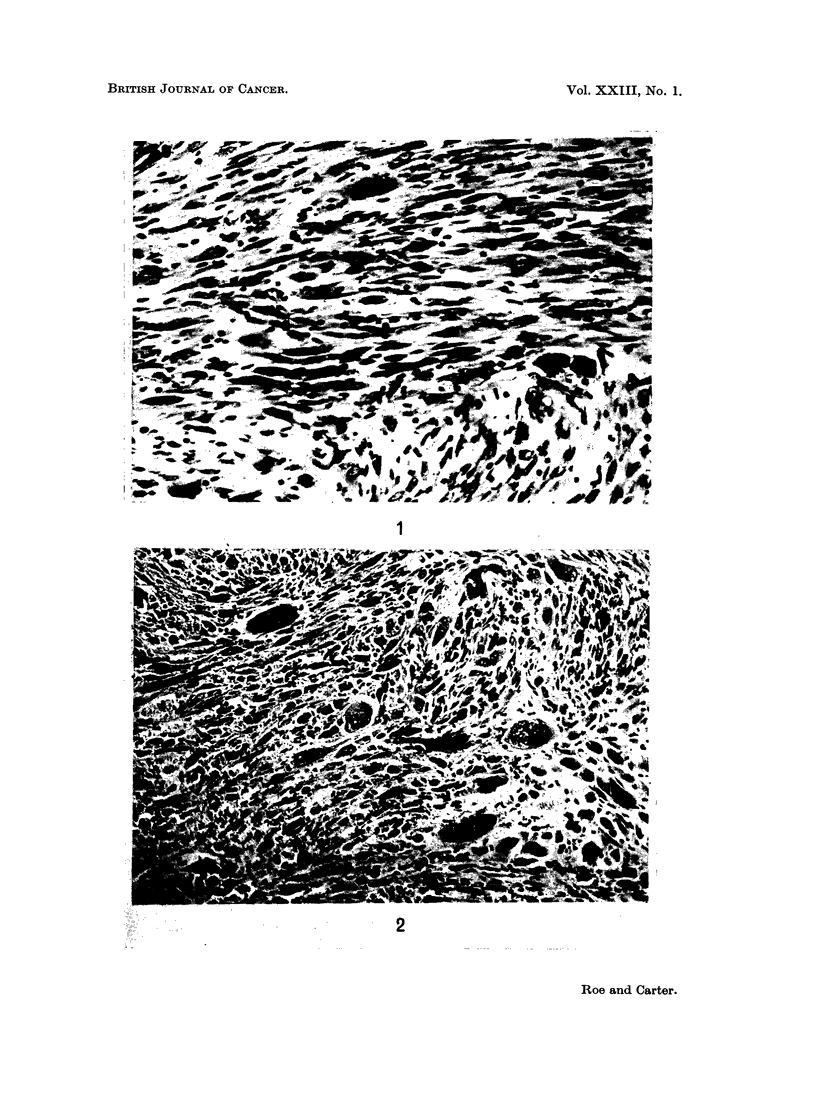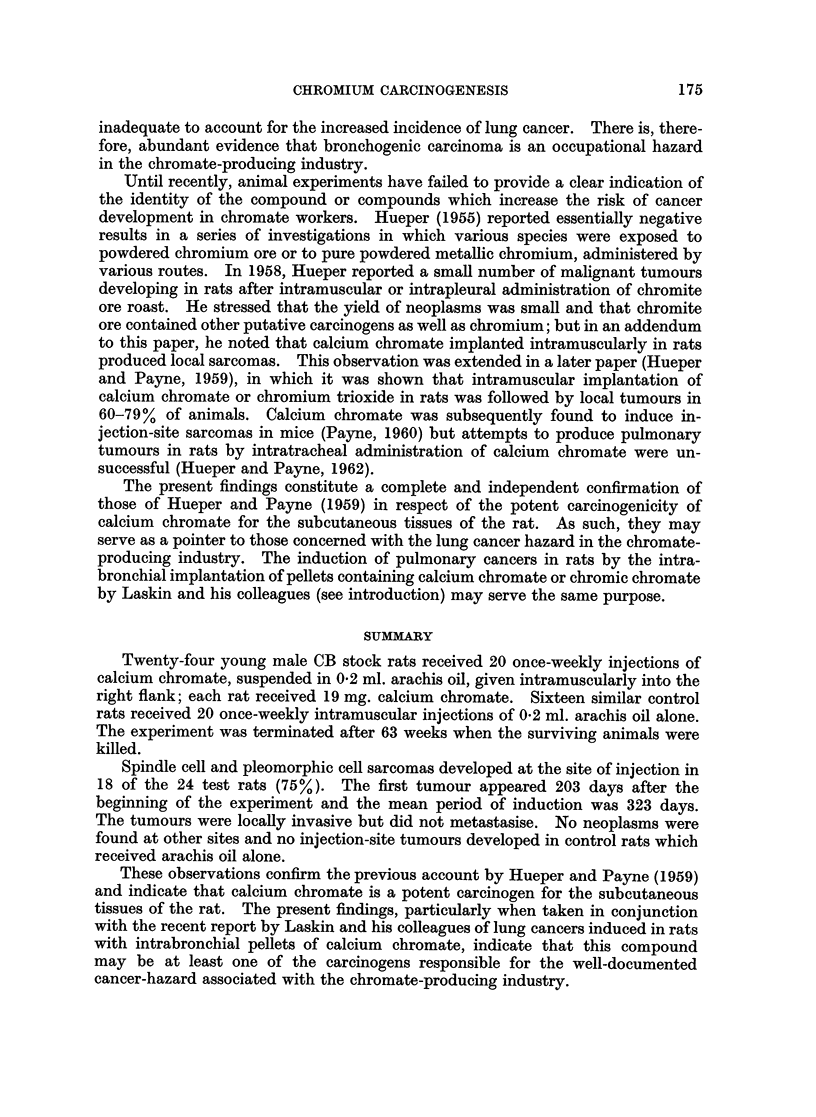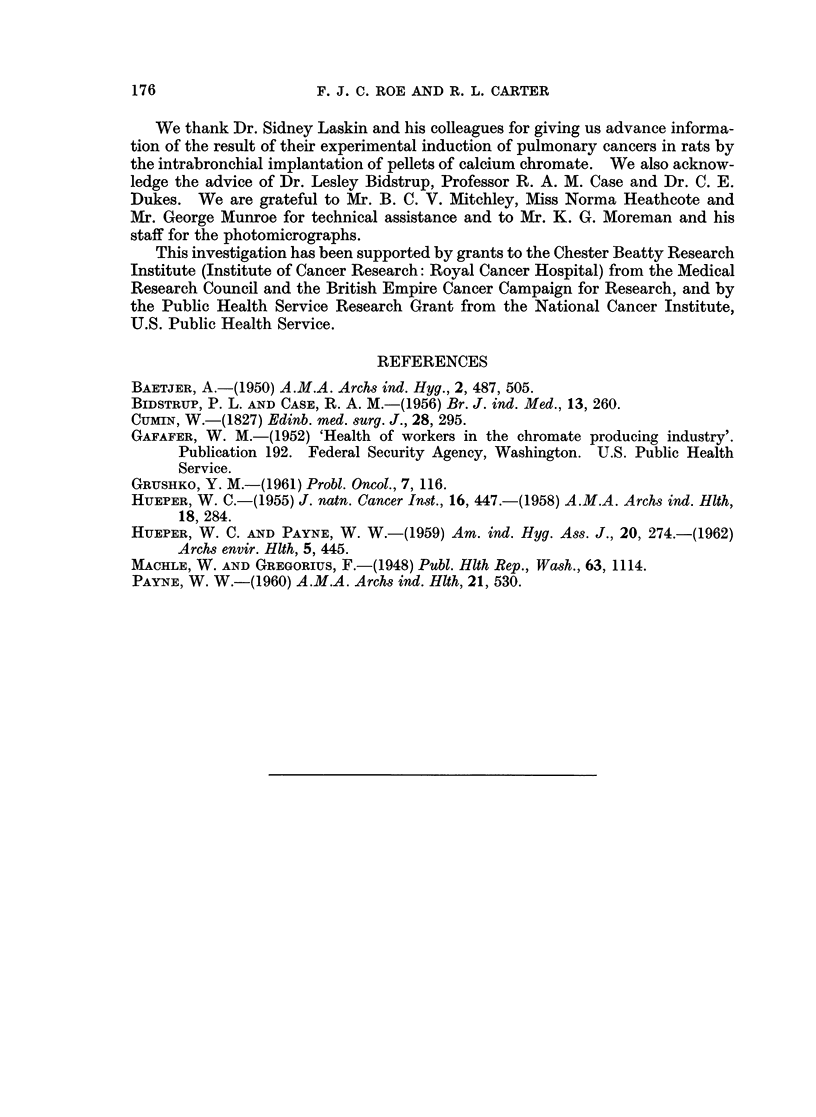# Chromium carcinogenesis: calcium chromate as a potent carcinogen for the subcutaneous tissues of the rat.

**DOI:** 10.1038/bjc.1969.25

**Published:** 1969-03

**Authors:** F. J. Roe, R. L. Carter

## Abstract

**Images:**


### CHROMIUM CARCINOGENESIS: CALCIUM CHROMATE AS A POTENT CARCINOGEN FOR THE SUBCUTANEOUS TISSUES OF THE RAT

F. J. C. ROE AND R. L. CARTER

*From the Chester Beatty Research Institute, Fulham Road, London, S.W.3*

Received for publication October 16, 1968

OVER a period of 15 years, we have investigated (at the request of the chromates-producing industry in Great Britain) the carcinogenicity of several different chromium-containing compounds by the technique of single or repeated intramuscular injection in rats and/or mice. The results of these tests were invariably negative until, at the independent suggestions of Dr. Sidney Laskin and Dr. Lesley Bidstrup, calcium chromate was examined in the same way.

The results of this experiment are now reported. Although a strikingly high yield of tumours was obtained, the present findings do no more than confirm those of Hueper and Payne (1959). But independent corroboration of their assertions in respect of calcium chromate seemed especially necessary in view of our inability to induce injection-site tumours with chromite ore roast, chromite roast residues or chromic chromate—all of which Hueper and Payne reported as carcinogenic on intramuscular (and also intrapleural) injection in rats (Hueper, 1958; Hueper and Payne, 1959).

The evidence for an association between exposure to chromates and lung cancer in man, critically reviewed by Gruschko (1961), provides the rationale for examining chromium-containing compounds for carcinogenicity. If certain industrial processes could be pin-pointed as especially hazardous, it would be possible to direct protective measures, currently being taken in all sectors, to those parts of the process which appear to be more likely to be associated with the risk of lung cancer. This might have the advantage to the industry of reducing the cost of ensuring that the risk is eliminated. Calcium chromate is formed in varying amounts when chromite ore is heated with limestone and sodium or potassium carbonate—the first step in processing the crude ore—and this would appear to be one important stage where potential carcinogens might be encountered.

The present findings should be considered alongisde those of Laskin and his colleagues who, at the meeting of the American Industrial Health Association in St. Louis in May 1968, reported the induction of squamous carcinomas of the lung in 6, and adenocarcinomas of the lung in 2, out of 100 rats exposed to calcium chromate in the form of a pellet attached to the bronchial mucosa.

### MATERIALS AND METHODS

*Experimental animals.*—Forty male CB stock rats, aged 5 to 6 weeks at the beginning of the experiment, were used. They were kept in metal cages, 5 in each, and fed on cubed diet No. 86 (Messrs Dixon Ltd., Ware, Herts) and water *ad libitum.*

*Calcium chromate.*—Calcium chromate was obtained from British Drug Houses, Ltd.

*Conduct of experiment*

The rats were divided into a test group of 24 animals and a control group of 16 animals. The *test group* received 20 once-weekly injections of calcium chromate suspended in 0·2 ml. arachis oil, given intramuscularly into the right flank. For the first 2 injections, the dose of calcium chromate was 5 mg. but signs of severe inflammation developed at the site of injection and the dose was reduced to 0·5 mg. for the remaining 18 weeks. The test animals thus received 19 mg. calcium chromate over a span of 20 weeks. The *control* group received 20 once-weekly injections of 0·2 ml. arachis oil, given intramuscularly into the right flank.

The rats were observed daily and examined for changes at the injection site. Animals were killed when they developed tumours or at the termination of the experiment at 63 weeks. Full post mortem examinations were carried out and tumours and tissues showing macroscopic abnormalities were removed and fixed in Bouin's solution. Paraffin sections were prepared at 5 $\mu$ and stained with haematoxylin and eosin.

### RESULTS

The results are shown in Table I. No systemic toxic effects attributable to calcium chromate were observed. During the period when injections were given, the injection sites became swollen and appeared tender but no ulceration of the overlying skin developed. The local swelling later subsided but there remained slight palpable thickening of the subcutaneous tissues at the injection site.

Tumours developed at the injection site in 18 of the 24 test animals (75%)—Table I. The first lesion appeared 203 days after the beginning of the experiment

TABLE I.—*Carcinogenesis in Rats Injected Subcutaneously with Calcium Chromate*

| | Days after the beginning of experiment | | | | | | |
|---|---|---|---|---|---|---|---|
| | 150 | 200 | 250 | 300 | 350 | 400 | 440 |
| *Test group* | | | | | | | |
| Survivors . . . . . . . . . | 24 | 24 | 24 | 22 | 17 | 9 | 0 |
| Cumulative total of tumour-bearing rats . . | 0 | 0 | 3 | 6 | 11 | 16 | 18 |
| Spindle cell sarcomas . . . . . . | 0 | 0 | 2 | 4 | 8 | 11 | 11 |
| Pleomorphic sarcomas . . . . . . | 0 | 0 | 1 | 2 | 3 | 5 | 7 |
| *Control group* | | | | | | | |
| Survivors . . . . . . . . . | 15 | 14 | 13 | 12 | 12 | 9 | 0 |
| Total of tumour-bearing rats . . . . . | 0 | 0 | 0 | 0 | 0 | 0 | 0 |

and the overall mean period of induction was 323 days. Once palpable, the tumours grew rapidly; all tumour-bearing rats were killed before their lesions began to ulcerate and the interval between the first detection of a tumour and the time when the rat had to be killed was, on average, 29 days. The neoplasms induced by calcium chromate were either spindle cell or pleomorphic cell sarcomata (Fig. 1 and 2). Some of the spindle cell lesions showed quite marked collagen formation. The pleomorphic tumours contained virtually no collagen fibres and were composed of bizarre binucleate and multinucleate cells, many of them with abnormal mitotic figures. No deposits of chromate were observed in or around the tumours. The sarcomas showed wide local invasion of the subcutaneous tissues, with extension into the muscles of the body wall, but no metastatic deposits were found.

The injection sites in the 6 test rats which did not develop local tumours, and in the 16 control animals injected with arachis oil alone, showed no specific features. There was no epidermal ulceration. The dermis contained increased amounts of fibrous tissue, infiltrates of macrophages and chronic inflammatory cells, and dilated lymphatic vessels. These changes were more prominent in rats injected with calcium chromate than in rats which were given arachis oil only.

No distant tumours developed in any rats in the test or control groups. Some animals from both groups showed bronchiectasis and cystic nephritis (sometimes accompanied by hydronephrosis) but there was no difference in the incidence, extent, or severity of these lesions between the test and control groups.

### DISCUSSION

In 1827, Cumin described ulcers and sloughs " of a peculiarly penetrating character " on the hands and forearms of Scottish workers who handled " bichromate of potass ". Similar cutaneous lesions were observed by later writers and perforating septal ulcers of the nose were reported in chromate workers by Delpech and Hillairet in 1869 (see Gafafer, 1952). Neither of these lesions was pre-neoplastic, no damage to other tissues following exposure to chromium-containing compounds was noted, and it was not until the 1930's that an association between exposure to chromates and the development of malignant disease—lung cancer—was suspected. German investigators (see reviews by Machle and Gregorius, 1948; Baetjer, 1950; Gafafer, 1952) were the first to recognise this association but their data were inadequate to calculate the risk involved; furthermore, the diagnosis of lung cancer was not always firmly established and some patients had been exposed to other known carcinogens. Machle and Gregorius (1948) conducted a survey of chromate workers in the United States and reported an exceptionally high risk of lung cancer in men engaged in the production of chromates and, to a lesser extent, of chrome pigments. No increase in cancer of other sites was found. Despite certain criticisms of the design of Machle and Gregorius's study, Baetjer (1950) confirmed their final conclusion and showed that lung cancer was not only more common but also occurred earlier in the chromate workers, the highest incidence being found in the 40–49 age group. In the United Kingdom, Bidstrup and Case (1956) also reported an increased incidence of mortality for cancer of the lung in the chromate-producing industry and noted a tendency for death due to bronchogenic carcinoma to occur disproportionately early. Important non-occupational factors such as the smoking habits and social class of the workers, and diagnostic bias, were examined and considered to be

---

### EXPLANATION OF PLATE

FIG. 1.—Spindle cell sarcoma arising at injection site of rat treated with calcium chromate. Tumour palpable at 273 days; rat killed at 300 days. H. and E. × 230.

FIG. 2.—Anaplastic sarcoma arising at injection site of rat treated with calcium chromate. Tumour palpable at 314 days; rat killed at 339 days. H. and E. × 180.

1

2

Roe and Carter.

inadequate to account for the increased incidence of lung cancer. There is, therefore, abundant evidence that bronchogenic carcinoma is an occupational hazard in the chromate-producing industry.

Until recently, animal experiments have failed to provide a clear indication of the identity of the compound or compounds which increase the risk of cancer development in chromate workers. Hueper (1955) reported essentially negative results in a series of investigations in which various species were exposed to powdered chromium ore or to pure powdered metallic chromium, administered by various routes. In 1958, Hueper reported a small number of malignant tumours developing in rats after intramuscular or intrapleural administration of chromite ore roast. He stressed that the yield of neoplasms was small and that chromite ore contained other putative carcinogens as well as chromium; but in an addendum to this paper, he noted that calcium chromate implanted intramuscularly in rats produced local sarcomas. This observation was extended in a later paper (Hueper and Payne, 1959), in which it was shown that intramuscular implantation of calcium chromate or chromium trioxide in rats was followed by local tumours in 60–79% of animals. Calcium chromate was subsequently found to induce injection-site sarcomas in mice (Payne, 1960) but attempts to produce pulmonary tumours in rats by intratracheal administration of calcium chromate were unsuccessful (Hueper and Payne, 1962).

The present findings constitute a complete and independent confirmation of those of Hueper and Payne (1959) in respect of the potent carcinogenicity of calcium chromate for the subcutaneous tissues of the rat. As such, they may serve as a pointer to those concerned with the lung cancer hazard in the chromate-producing industry. The induction of pulmonary cancers in rats by the intrabronchial implantation of pellets containing calcium chromate or chromic chromate by Laskin and his colleagues (see introduction) may serve the same purpose.

### SUMMARY

Twenty-four young male CB stock rats received 20 once-weekly injections of calcium chromate, suspended in 0·2 ml. arachis oil, given intramuscularly into the right flank; each rat received 19 mg. calcium chromate. Sixteen similar control rats received 20 once-weekly intramuscular injections of 0·2 ml. arachis oil alone. The experiment was terminated after 63 weeks when the surviving animals were killed.

Spindle cell and pleomorphic cell sarcomas developed at the site of injection in 18 of the 24 test rats (75%). The first tumour appeared 203 days after the beginning of the experiment and the mean period of induction was 323 days. The tumours were locally invasive but did not metastasise. No neoplasms were found at other sites and no injection-site tumours developed in control rats which received arachis oil alone.

These observations confirm the previous account by Hueper and Payne (1959) and indicate that calcium chromate is a potent carcinogen for the subcutaneous tissues of the rat. The present findings, particularly when taken in conjunction with the recent report by Laskin and his colleagues of lung cancers induced in rats with intrabronchial pellets of calcium chromate, indicate that this compound may be at least one of the carcinogens responsible for the well-documented cancer-hazard associated with the chromate-producing industry.

We thank Dr. Sidney Laskin and his colleagues for giving us advance information of the result of their experimental induction of pulmonary cancers in rats by the intrabronchial implantation of pellets of calcium chromate. We also acknowledge the advice of Dr. Lesley Bidstrup, Professor R. A. M. Case and Dr. C. E. Dukes. We are grateful to Mr. B. C. V. Mitchley, Miss Norma Heathcote and Mr. George Munroe for technical assistance and to Mr. K. G. Moreman and his staff for the photomicrographs.

This investigation has been supported by grants to the Chester Beatty Research Institute (Institute of Cancer Research: Royal Cancer Hospital) from the Medical Research Council and the British Empire Cancer Campaign for Research, and by the Public Health Service Research Grant from the National Cancer Institute, U.S. Public Health Service.

### REFERENCES

BAETJER, A.—(1950) *A.M.A. Archs ind. Hyg.*, **2**, 487, 505.

BIDSTRUP, P. L. AND CASE, R. A. M.—(1956) *Br. J. ind. Med.*, **13**, 260.

CUMIN, W.—(1827) *Edinb. med. surg. J.*, **28**, 295.

GAFAFER, W. M.—(1952) 'Health of workers in the chromate producing industry'. Publication 192. Federal Security Agency, Washington. U.S. Public Health Service.

GRUSHKO, Y. M.—(1961) *Probl. Oncol.*, **7**, 116.

HUEPER, W. C.—(1955) *J. natn. Cancer Inst.*, **16**, 447.—(1958) *A.M.A. Archs ind. Hlth*, **18**, 284.

HUEPER, W. C. AND PAYNE, W. W.—(1959) *Am. ind. Hyg. Ass. J.*, **20**, 274.—(1962) *Archs envir. Hlth*, **5**, 445.

MACHLE, W. AND GREGORIUS, F.—(1948) *Publ. Hlth Rep., Wash.*, **63**, 1114.

PAYNE, W. W.—(1960) *A.M.A. Archs ind. Hlth*, **21**, 530.